# Exploring large language model for next generation of artificial intelligence in ophthalmology

**DOI:** 10.3389/fmed.2023.1291404

**Published:** 2023-11-23

**Authors:** Kai Jin, Lu Yuan, Hongkang Wu, Andrzej Grzybowski, Juan Ye

**Affiliations:** ^1^Eye Center, The Second Affiliated Hospital, School of Medicine, Zhejiang University, Hangzhou, China; ^2^Department of Ophthalmology, The Children's Hospital, Zhejiang University School of Medicine, National Clinical Research Center for Child Health, Hangzhou, China; ^3^Institute for Research in Ophthalmology, Foundation for Ophthalmology Development, Poznan, Poland

**Keywords:** artificial intelligence, large language model, ChatGPT, ophthalmology, diagnostic accuracy and efficacy

## Abstract

In recent years, ophthalmology has advanced significantly, thanks to rapid progress in artificial intelligence (AI) technologies. Large language models (LLMs) like ChatGPT have emerged as powerful tools for natural language processing. This paper finally includes 108 studies, and explores LLMs’ potential in the next generation of AI in ophthalmology. The results encompass a diverse range of studies in the field of ophthalmology, highlighting the versatile applications of LLMs. Subfields encompass general ophthalmology, retinal diseases, anterior segment diseases, glaucoma, and ophthalmic plastics. Results show LLMs’ competence in generating informative and contextually relevant responses, potentially reducing diagnostic errors and improving patient outcomes. Overall, this study highlights LLMs’ promising role in shaping AI’s future in ophthalmology. By leveraging AI, ophthalmologists can access a wealth of information, enhance diagnostic accuracy, and provide better patient care. Despite challenges, continued AI advancements and ongoing research will pave the way for the next generation of AI-assisted ophthalmic practices.

## Introduction

**Figure 1:**
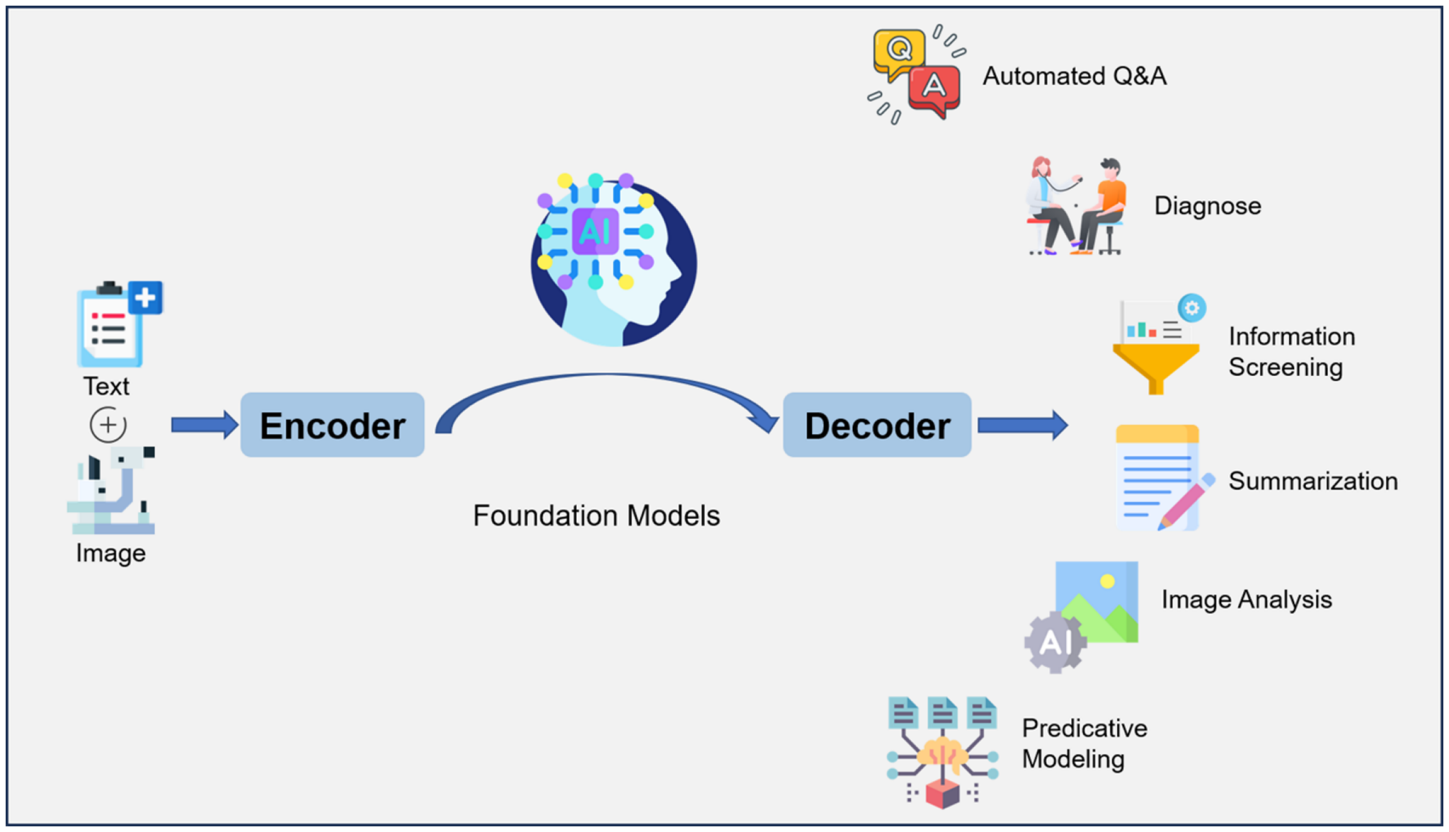
Workflow of large language model (LLM) for artificial intelligence (AI) in ophthalmology. Text (symptoms, medical history, etc.) and images (Optical coherence tomography, Fundus fluorescein angiography, etc.) are encoded and fed into a model that has been trained on a large amount of data, which can decode the relevant information required. LLM applications include automated question-answering, diagnose, information screening, summarization, image analysis, predictive modeling.

The history of artificial intelligence (AI) in medicine dates back to the 1950s when researchers began to explore the use of computers to analyze medical data and make diagnostic decisions. However, past methods had limitations in accuracy and speed and still could not analyze unstructured medical data (1). Natural Language Processing (NLP) is a subfield of AI that focuses on enabling computers to understand, interpret, and generate human language. It involves the development of algorithms and models that can process and analyze unstructured text data. Large Language Models (LLM) refer to advanced artificial intelligence models, such as GPT-3 (Generative Pre-trained Transformer 3), that are built on transformer architecture. The transformer architecture is a deep learning model that efficiently captures context and dependencies in sequential data, making it a fundamental choice for natural language processing tasks and beyond. These models are trained on massive amounts of text data from the internet, enabling them to generate human-like text and perform a wide range of NLP tasks with remarkable accuracy and versatility. ChatGPT builds on the capabilities of large language models to generate coherent and contextually relevant responses, making it well-suited for chatbot applications. It is designed to generate human-like responses to a wide range of prompts and questions and may enhance healthcare delivery and patients’ quality of life (Figure 1) (2). The use of LLMs in healthcare offers several potential benefits.

ChatGPT and LLMs can be applied in various ways. They can serve as clinical documentation aids, helping with administrative tasks such as clinic scheduling, medical coding for billing, and generating preauthorization letters (3). LLMs can also be used as summarization tools, improving communication with patients and assisting in clinical trials. They can make processes such as curriculum design, testing of knowledge base, and continuing medical education more dynamic (4). LLMs can reduce the burden of administrative tasks for healthcare professionals, save time, and improve efficiency. They also have the potential to provide valuable clinical insights and support decision-making (5). This capability may help ophthalmologists enabling evidence-based decision-making and revolutionizing various aspects of eye care and research.

## Method of literature search

For this review, we followed the Preferred Reporting Items for Systematic Reviews and Meta-Analyzes (PRISMA) guidelines.

### Study selection and search strategy

We conducted a comprehensive literature search following the PRISMA guidelines. Searches were performed on PubMed and Google Scholar databases, spanning from January 2016 to June 2023. Keywords were selected from two distinct categories: ophthalmology-related terms (ophthalmology, eye diseases, eye disorders) and large language model-related terms (large language models, ChatGPT, natural language processing, chatbots). The search strategy involved the use of the following keywords: (“Ophthalmology” OR “Eye Diseases” OR “Eye Disorders”) AND (“Large Language Models” OR “ChatGPT” OR “Natural Language Processing” OR “Chatbots”). The terms from each category were cross-referenced independently with terms from the other category.

### Inclusion and exclusion criteria

We established specific inclusion criteria for article selection. The publication period considered research from January 2016 to June 2023 to ensure the inclusion of up-to-date findings. Initially, 6,130 articles were identified through titles and abstracts. We prioritized research quality and the application of Large Language Models (LLMs) in our selection process. Additionally, articles published prior to 2016 were included for historical context and those pertinent to closely related topics.

In the meantime, studies meeting the following criteria will be excluded: (1) duplicate literature previously included in the review, (2) irrelevant topics, where the article is unrelated to ophthalmology or the application of the large language model, (3) conference abstracts, and (4) non-original research, such as editorials, case reports or commentaries.

### Language considerations

A comprehensive review was conducted primarily on English-language articles, totaling 6,130 papers. Furthermore, we evaluated 14 papers predominantly published in Chinese. For articles in languages such as French, Spanish, and German, we assessed their abstracts. This multilingual approach allowed us to comprehensively evaluate the literature. The primary inclusion criterion required research to specifically address the application of AI in ophthalmology and demonstrate a certain level of perceived quality.

### Data extraction and analysis

Following a rigorous selection process, relevant data were extracted and analyzed from the selected articles. Key themes, trends, advancements, and challenges related to the utilization of LLMs in ophthalmology were systematically synthesized.

**Figure 2:**
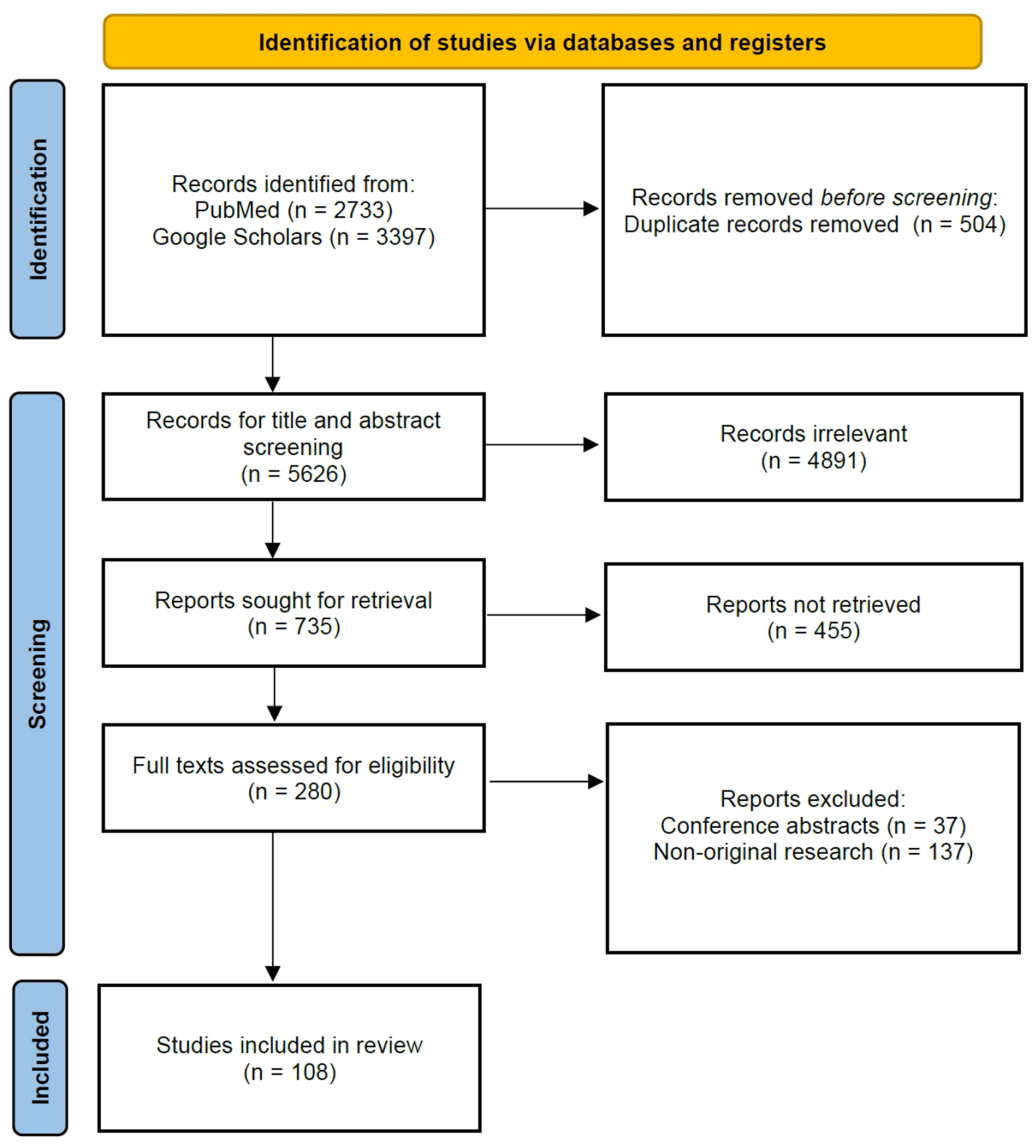
PRISMA 2020 flow diagram for this systematic review.

In accordance with the PRISMA guidelines, this review adhered to a structured and rigorous approach, encompassing a comprehensive literature search, meticulous inclusion criteria, language considerations, and thorough data extraction (Figure 2). A total of 108 articles were independently screened for eligibility by two reviewers (Kai Jin and Lu Yuan), including assessments of titles and abstracts, followed by full-text review. Any disagreements were resolved through discussion with a third author (Juan Ye). Ultimately, 108 studies were included in the review.

## Results

**Figure 3:**
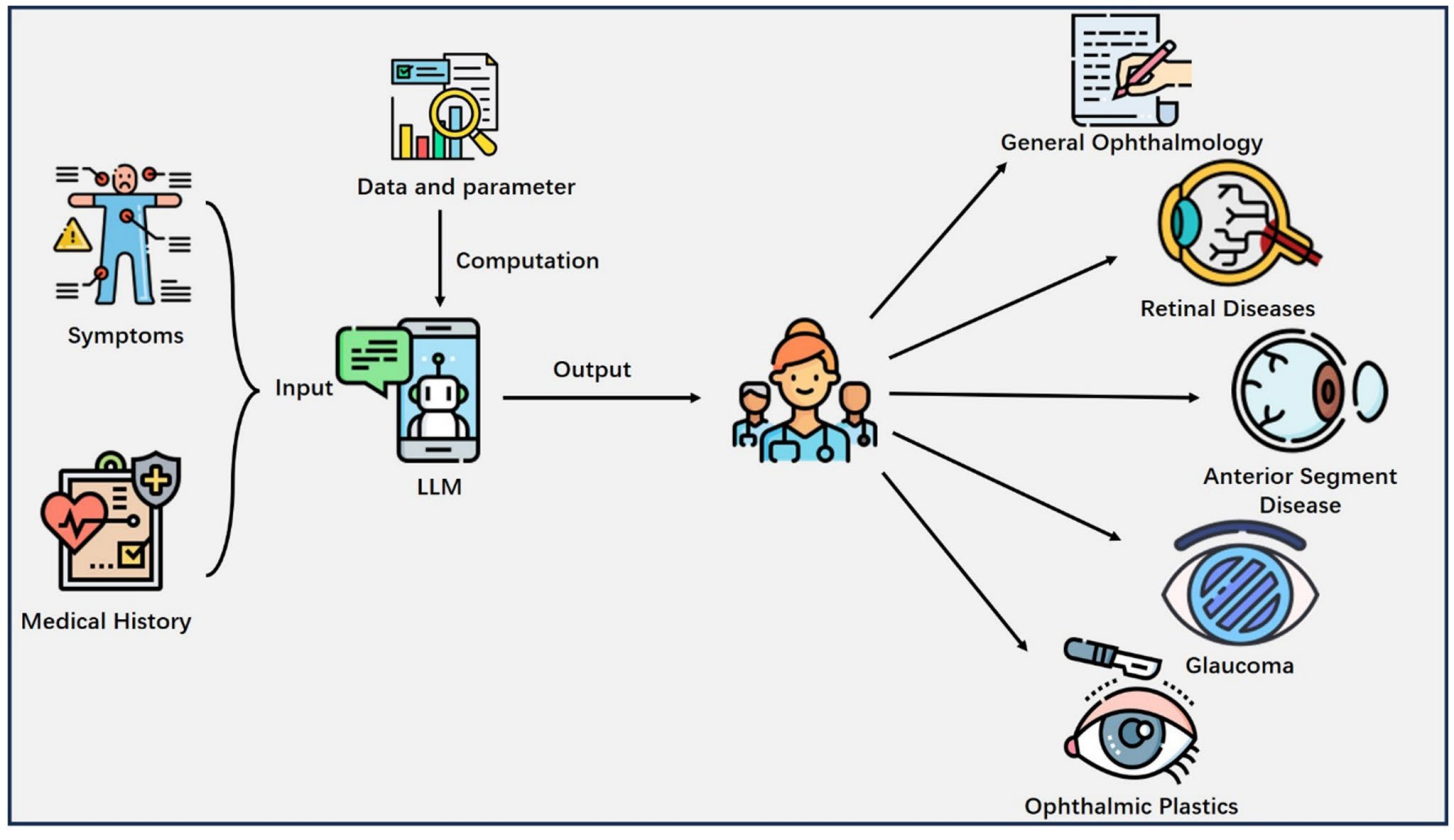
Major applications of LLM in Ophthalmology. The patient’s information like symptoms, medical history and other health-related details are inputted into the LLM, which outputs valuable clinical insights to the physician and helps him or her make decisions.

We finally included 108 studies. The results (Table 1) encompass a diverse range of studies in the field of ophthalmology, highlighting the versatile applications of LLMs. The results reflect a wide spectrum of LLM applications, and subfields of interest in ophthalmology. They showcase the versatility of LLMs in addressing various aspects of automated question-answering (55 studies), diagnose (5 studies), information screening (27 studies), summarization (5 studies), image analysis (5 studies), predictive modeling (11 studies). Subfields encompass general ophthalmology (38 studies), retinal diseases (32 studies), anterior segment diseases (27 studies), glaucoma (6 studies), and ophthalmic plastics (5 studies) (Figure 3).

**Table 1:** Summary of representative current studies using LLM in ophthalmology.

Reference	Year	Publication	Subspeciality	Aim	Application	Approaches
Lin et al. (6)	2023	Eye	General ophthalmology	To compare the performance on a practice ophthalmology written examination	Automated question-answering	GPT-3.5, GPT-4
Antaki et al. (7)	2023	Ophthalmology Science	General ophthalmology	To evaluate the performance on ophthalmology questions	Automated question-answering	ChatGPT
Cai et al. (8)	2023	American Journal of Ophthalmology	General ophthalmology	To compare the performance on ophthalmology board-style questions.	Automated question-answering	Bing Chat, ChatGPT 3.5, and ChatGPT 4.0,
Mihalache et al. (9)	2023	JAMA Ophthalmology	General ophthalmology	To assess the performance on board certification exam in ophthalmology	Automated question-answering	ChatGPT
Bernstein et al. (10)	2023	JAMA Network Open	General ophthalmology	To generate ophthalmology advice	Automated question-answering	ChatGPT version 3.5
Ali et al. (11)	2023	Ophthalmic Plast Reconstr Surg	Lacrimal drainage disorders	To response to lacrimal drainage disorders	Automated question-answering	ChatGPT
Tsui et al. (12)	2023	Eye	Posterior vitreous detachment, retinal tear and detachment, ocular surface disease, exudative age-related macular degeneration (eAMD), and post-intravitreal injection pain and redness	To response to common ocular symptoms	Automated question-answering	ChatGPT
Potapenko et al. (13)	2023	Acta Ophthalmologica	Retinal diseases	To evaluate accuracy on patient information	Automated question-answering	ChatGPT
Momenaei et al. (14)	2023	Ophthalmology Retina	Retinal diseases	To evaluate the appropriateness and readability of the medical knowledge	Automated question-answering	ChatGPT-4
Waisberg et al. (15)	2023	Irish Journal of Medical Science	Anterior ischemic optic neuropathy	Fundus image analysis	Image analysis	GPT-4
Hu et al. (16)	2022	Transl Vis Sci Technol.	Glaucoma	To Predict Glaucoma Progression Requiring Surgery	Predictive Modeling	Pre-trained Transformers
Lee et al. (17)	2023	Ophthalmic Res	General ophthalmology	To assign procedural codes based on the surgical report	Predictive Modeling	Bidirectional Encoder Representations from Transformers (BERT)
Liu et al. (18)	2023	AMIA	Retinal vascular disease	To provide a diagnosis based on FFA reports	Summarization	GPT3.5-Turbo
Yu et al. (19)	2022	BMC Medical Informatics and Decision Making	Diabetic retinopathy	To Identify diabetic retinopathy-related clinical concepts and their attributes	Information screening	NLP(Extraction, Named entity recognition), DL, Pre-trained Transfomers
Valentín-Bravo et al. (20)	2023	Arch Soc Esp Oftalmol.	Vitreoretinal disease	To write a scientific article	Information screening	ChatGPT, DALL-E 2
Singh et al. (4)	2023	Clin Exp Ophthamol.	Dry eye disease	To conduct a literature review	Information screening	ChatGPT
Singh et al. (21)	2023	Seminars in Ophthalmology	Cornea, retina, glaucoma, pediatric ophthalmology, neuroophthalmology, and ophthalmic plastics surgery	To construct ophthalmic discharge summaries and operative notes	Information screening	ChatGPT
Rasmussen et al. (22)	2023	Graefe’s archive for clinical and experimental ophthalmology	Vernal keratoconjunctivitis	To provided responses to patient and parent questions	Automated question-answering	ChatGPT
lim et al. (23)	2023	Ebiomedicine	Myopia	To deliver accurate responses to common myopia-related query	Automated question-answering	ChatGPT-3.5, ChatGPT-4.0, and Google Bard
Waisberg et al. (24)	2023	Annals of Biomedical Engineering	General ophthalmology	To write ophthalmic operative notes	Information screening	GPT-4

### General ophthalmology

The application of LLMs in ophthalmology is a rapidly growing field with promising potential, encompassing various aspects of patient care and clinical workflows. LLMs can analyze general ophthalmology patient data and medical records to recommend personalized diagnosis and treatment plans for individuals with specific eye conditions. Chatbots integrated with electronic health record (EHR) systems can access patient information to provide context-aware responses and support clinical decision-making.

The majority of current NLP applications in ophthalmology focus on extracting specific text, such as visual acuity, from free-text notes for the purposes of quantitative analysis (25). NLP also offers opportunities to develop search engines for data within free-text notes, clean notes, automated question-answering, and translating ophthalmology notes for other specialties or for patients. Low vision rehabilitation improves quality of life for visually impaired patients, free-text progress notes within the EHR using NLP provide valuable information relevant to predicting patients’ visual prognosis (26). NLP with unstructured clinician notes supports low vision and blind rehabilitation for war veterans with traumatic brain injury based on veterans’ needs rather than system-level factors (27, 28). This suggests that AI with NLP may be particularly important for the performance of predictive models in ophthalmology. Given the potential of LLMs in healthcare and the increasing reliance of patients on online information, it is important to evaluate the quality of chatbot-generated advice and compare it with human-written advice from ophthalmologists. The panel of ophthalmologists had a 61.3% accuracy in distinguishing between chatbot and human responses (10).

As chatbot technology is continually evolving, there are additional applications in general ophthalmology. The researchers evaluated the ability of the ChatGPT to respond to ocular symptoms by scripting 10 prompts reflective of common patient messages relating to various ocular conditions (12). These conditions included posterior vitreous detachment, retinal tear and detachment, ocular surface disease, exudative age-related macular degeneration (eAMD), and post-intravitreal injection pain and redness. The abilities of ChatGPT in constructing discharge summaries and operative notes were evaluated through a study conducted by Swati et al. (21). The study found that ChatGPT was able to construct ophthalmic discharge summaries and operative notes in a matter of seconds, with tailored responses based on the quality of inputs given. However, there were some limitations such as the presence of generic text and factual inaccuracies in some responses. The authors suggest that ChatGPT can be utilized to minimize the time spent on discharge summaries and improve patient care, but it should be used with caution and human verification. Another study aimed to assess the performance of an AI chatbot, ChatGPT, in answering practice questions for ophthalmology board certification examinations (9). ChatGPT correctly answered 46.4% of the questions, with the best performance in the category of general medicine (79%) and the poorest in retina and vitreous (0%). ChatGPT provided explanations and additional insight for 63% of questions but selected the same multiple-choice response as the most common answer provided by ophthalmology trainees only 44% of the time. The researchers compared the performance of several generative AI models on the ophthalmology board-style questions (6–8), including Bing Chat (Microsoft), ChatGPT 3.5 and 4.0 (OpenAI). Performance was compared with that of human respondents. Results showed that ChatGPT-4.0 and Bing Chat performed comparably to human respondents.

Existing electronic differential diagnosis support tools, like the Isabel Pro Differential Diagnosis Generator, have limitations in terms of structured input and context-specific language processing. In one study, ChatGPT identified the correct diagnosis in 9 out of 10 cases and had the correct diagnosis listed in all 10 of its lists of differentials (29). Isabel, on the other hand, identified only 1 out of 10 provisional diagnoses correctly, but included the correct diagnosis in 7 out of 10 of its differential diagnosis lists. The median position of the correct diagnosis in the ranked differential lists was 1.0 for ChatGPT versus 5.5 for Isabel.

### Retinal diseases

Some studies evaluate the accuracy of an AI-based chatbot in providing patient information on common retinal diseases, including AMD, diabetic retinopathy (DR), retinal vein occlusion, retinal artery occlusion, and central serous chorioretinopathy.

In healthcare settings, when patients provide information about their medical history, symptoms, or other health-related details, there is the potential for miscommunication or misalignment between the patient’s perspective and the physician’s understanding of the situation. Traditional methods of obtaining patient information may lead to dissatisfaction if the information obtained misaligns with the physician’s information (30). ChatGPT can improve patient satisfaction in terms of information provision by providing accurate and well-formulated responses to various topics, including common retinal diseases (13). This accessibility can be particularly beneficial when ophthalmologists are not readily available. Among retinal diseases, DR is a leading cause of blindness in adults, and there is increasing interest in developing AI technologies to detect DR using EHRs. Most AI-based DR diagnoses are focused on medical images, but there is limited research exploring the lesion-related information captured in the free text image reports. In Yu et al. (19) study, two state-of-the-art transformer-based NLP models, including BERT and RoBERTa, were examined and compared with a recurrent neural network implemented using Long short-term memory (LSTM) to extract DR-related concepts from clinical narratives. The results show that for concept extraction, the BERT model pretrained with the MIMIC III dataset outperformed other models, achieving the highest performance with F1-scores of 0.9503 and 0.9645 for strict and lenient evaluations, respectively. The findings of this study could have a significant impact on the development of clinical decision support systems for DR diagnoses.

### Anterior segment disease

Anterior segment vision-threatening disease included the diagnosis of corneal ulcer, iridocyclitis, hyphema, anterior scleritis, or scleritis with corneal involvement. Patients with anterior segment diseases present a diagnostic challenge for many primary care physicians. The researchers developed a decision support tool to predict vision-threatening anterior segment disease using primary clinical notes based on NLP (31). The ultimate prediction model exhibited an area under the curve (AUC) of 0.72, with a 95% confidence interval ranging from 0.67 to 0.77. Using a threshold that achieved a sensitivity of 90%, the model demonstrated a specificity of 30%, a positive predictive value of 5.8%, and a high negative predictive value of 99%. One study evaluates the accuracy of responses provided by the ChatGPT to patient and parent questions on vernal keratoconjunctivitis (VKC), a complex and recurring disease primarily affecting children (22). The researchers formulated questions in four categories and assessed the chatbot’s responses for information accuracy. The chatbot was found to provide both relevant and inaccurate statements. Inaccurate statements were particularly observed regarding treatment and potential side effects of medications. A comparative analysis of the performance of three LLMs, namely ChatGPT-3.5, ChatGPT-4.0, and Google Bard, was conducted in delivering accurate and comprehensive responses to common myopia-related queries. ChatGPT-4.0 demonstrated the highest accuracy, with 80.6% of responses rated as ‘good’, compared to 61.3% in ChatGPT-3.5 and 54.8% in Google Bard (23).

### Glaucoma

Previous studies have developed predictive models for glaucoma progression, but uncertainty remains on how to integrate the information in free-text clinical notes, which contain valuable clinical information (32). Some studies aim to predict glaucoma progression requiring surgery using deep learning approaches on EHRs and natural language processing of clinical free-text notes. Sunil et al. presents an artificial intelligence approach to predict near-term glaucoma progression using clinical free-text notes and data from electronic health records (33). The authors developed models that combined structured data and text inputs to predict whether a glaucoma patient would require surgery within the following year. The model incorporating both structured clinical features and free-text features achieved the highest performance with an AUC of 0.899 and an F1 score of 0.745. Another study aims to fill the gap by developing a deep learning predictive model for glaucoma progression using both structured clinical data and natural language processing of clinical free-text notes from EHRs. The combination model showed the best AUC (0.731), followed by the text model (0.697) and the structured model (0.658) (34). Hu et al. (16) explored the use of transformer-based language models, specifically Bidirectional Encoder Representations from Transformers (BERT), to predict glaucoma progression requiring surgery using clinical free-text notes from EHRs. The results showed that the BERT models outperformed an ophthalmologist’s review of clinical notes in predicting glaucoma progression. Michelle et al. (35) utilized an automated pipeline for data extraction from EHRs to evaluate the real-world outcomes of glaucoma surgeries, tube shunt surgery had a higher risk of failure (Baerveldt: Hazard Ratio (HR) 1.44, 95% CI 1.02 to 2.02; Ahmed: HR 2.01, 95% CI 1.28 to 3.17).

### Ophthalmic plastics

In the study conducted by Mohammad et al. (11), ChatGPT’s performance in providing information about primary acquired nasolacrimal duct obstruction and congenital nasolacrimal duct obstruction was evaluated. Regarding insights into the history and effectiveness of dacryocystorhinostomy surgery, ChatGPT was tested on this specific topic. Agreement among the three observers was high (95%) in grading the responses. The responses of ChatGPT were graded as correct for only 40% of the prompts, partially correct in 35%, and outright factually incorrect in 25%. Hence, some degree of factual inaccuracy was present in 60% of the responses, if we consider the partially correct responses.

## Discussion

The newer generation of GPT models, exemplified by GPT-3 and beyond, differs from their predecessors through significantly larger model sizes, improved performance on various language tasks, enhanced few-shot learning abilities, and increased versatility, while also necessitating more substantial computational resources and raising ethical considerations.

### Strengths

AI technology, such as online chat-based AI language models, has the potential to assist clinical workflows and augment patient education and communication about common ophthalmology diseases prevention queries (Table 1). GPT’s medical subspecialty capabilities have improved significantly from GPT-3 to GPT-4. Both LLMs struggled with image-based and higher-order ophthalmology questions, perhaps reflecting the importance of visual analysis in ophthalmology. Given the ongoing advances in computer vision, it may be possible to address this limitation in future LLMs. There is room for improvement in medical conversational agents, as all models exhibited instances of hallucination, incorrect justification, or non-logical reasoning (36). Although ChatGPT 4.0 has demonstrated remarkable capabilities in a variety of domains, the presence of these errors raises concerns about the reliability of the system, especially in critical clinical decision making.

Ophthalmologists are starting to use ChatGPT to help with paperwork such as scientific articles, discharge summaries and operative notes (15, 24, 37). The scientific accuracy and reliability on certain topics were not sufficient to automatically generate scientifically rigorous articles. This was also objected to by some ophthalmologists (38). Firstly, operative notes are not general descriptions of surgical procedures and a specific patient has its own unique characteristics. Secondly, operative notes are legal documents and the surgeon is responsible for the accuracy and completeness of the notes. Thirdly, there is no evidence that GPT-4 can accurately capture the unique aspects of individual cases in the real world, such as intraoperative complications. Finally, the writing of operative notes requires a degree of clinical decision-making and clinical judgment that cannot be automated.

In a recent development, ChatGPT has emerged as an author or co-author of scientific papers in the field of ophthalmology (39, 40). This innovative inclusion has sparked discussions and garnered attention from the scientific community. The presence of ChatGPT as an author in scientific research reflects the evolving landscape of artificial intelligence’s involvement in various domains, including ophthalmology, opening avenues for new perspectives and collaborative contributions.

### Challenges

Despite the promising future, integrating LLMs into ophthalmology also poses several challenges that need to be addressed. Firstly, ensuring patient data privacy and maintaining the security of sensitive medical information will be critical (41). These models require vast amounts of data to achieve their potential, but data-sharing must be conducted responsibly and in compliance with strict ethical and legal guidelines (42, 43).

Another significant challenge is the potential for bias in the data used to train these language models (44). If the data used for training is not diverse enough, the models may exhibit biases that can lead to inaccurate or unfair recommendations, particularly when dealing with underrepresented populations. Efforts must be made to identify and mitigate these biases to ensure equitable and reliable outcomes for all patients.

Furthermore, there may be resistance or skepticism among some healthcare professionals towards adopting AI-driven technologies like LLMs. It will be crucial to address these concerns, provide proper training, and foster a collaborative environment where human experts and AI work together synergistically (45).

The interpretability and explainability of the decisions made by these models are another challenge. As they are often considered “black boxes,” “understanding the reasoning behind their recommendations can be difficult,” leading to potential mistrust from clinicians and patients (46). Developing methods to make the models more transparent and explainable will be essential for their widespread acceptance and adoption (47).

Lastly, the rapidly evolving nature of AI and language model technologies demands continuous updates and improvements. Staying up-to-date with the latest advancements and incorporating new knowledge into the models is essential to maintain their accuracy and relevance in the ever-changing field of ophthalmology.

While LLMs like ChatGPT offer tremendous potential in ophthalmology, addressing the challenges of AI hallucination and misinformation is paramount. It is essential to consider the broader societal implications, including patient trust, medical liability, ethical concerns, scientific integrity, health disparities, and regulatory oversight when integrating AI into ophthalmic practices. Responsible AI implementation and continuous monitoring are essential to harness the benefits of AI while minimizing potential risks. One concern in the use of LLMs for medical applications is the lack of reproducibility, as these generative models may not consistently provide the same answers, potentially impacting the reliability of their outputs in clinical settings. Addressing these challenges will be essential to fully realize the potential benefits of large language models in ophthalmology and to ensure their responsible and ethical implementation in patient care (48).

### Future perspectives

The future perspectives of LLMs in ophthalmology hold tremendous promise for transforming the landscape of eye care and research (49). These advanced language models, powered by AI and NLP, are poised to revolutionize how ophthalmologists diagnose, treat, and manage various eye conditions. LLMs can be integrated with image analysis techniques to create multimodal AI systems. These systems can process both textual and visual information, enhancing their capabilities in ophthalmology. For instance, LLMs can analyze textual patient records and medical literature, while image analysis algorithms can interpret medical images such as fundus photographs. Through their ability to analyze vast amounts of medical literature, patient data, and diagnostic images, these models can provide more accurate and timely diagnoses, personalized treatment plans, and even predict disease progression. The combination of LLMs and image analysis can lead to more efficient and accurate decision-making in ophthalmic practice. Additionally, LLMs can be used as tools to support communication and knowledge exchange in the following ways. While LLMs themselves do not directly facilitate communication like human interaction, their capabilities can enhance and streamline information exchange and knowledge sharing among eye care professionals worldwide. As research and development in this field continue to progress, we can expect these language models to become indispensable tools that enhance efficiency, accessibility, and ultimately improve patient outcomes in ophthalmology.

### Limitations

This review acknowledges several potential limitations that may have affected the comprehensiveness and potential bias of the literature search and selection process. These limitations include publication bias, language bias due to the focus on English-language studies, potential database selection bias, the possibility of excluding relevant studies due to search term restrictions, the limited date range, and the predefined exclusion criteria that may have omitted relevant research. The review also recognizes the potential for missed references and acknowledges the subjectivity in reviewer bias, which could impact study inclusion. Moreover, the review underscores the importance of addressing these limitations to ensure a more comprehensive and balanced assessment of the field of AI in ophthalmology. Despite these potential constraints, the review provides valuable insights into the applications and challenges of AI in ophthalmology, but readers should consider these limitations when interpreting the findings and drawing conclusions from the review.

## Author contributions

KJ: Conceptualization, Data curation, Funding acquisition, Investigation, Methodology, Writing – original draft, Writing – review & editing. LY: Data curation, Formal analysis, Investigation, Methodology, Writing – original draft. HW: Formal analysis, Software, Validation, Writing – review & editing. AG: Supervision, Validation, Visualization, Writing – review & editing. JY: Conceptualization, Funding acquisition, Project administration, Resources, Supervision, Validation, Visualization, Writing – review & editing.
